# Acupuncture combined with antidepressants for mild-to-moderate depressive disorders: a systematic review with meta-analysis and trial sequential analysis

**DOI:** 10.3389/fneur.2025.1636589

**Published:** 2025-12-12

**Authors:** Yingdong Wang, Yi Yu, Shike Zhang, Qiang Xi, Jiwen Qiu, Xinju Li, Mingxing Zhang, Yi Guo

**Affiliations:** 1 School of Acupuncture and Tuina, Tianjin University of Traditional Chinese Medicine, Tianjin, China; 2 School of Traditional Chinese Medicine, Tianjin University of Traditional Chinese Medicine, Tianjin, China; 3 School of Medical Technology, Tianjin University of Traditional Chinese Medicine, Tianjin, China; 4 School of Integrated Traditional Chinese and Western Medicine, Tianjin University of Traditional Chinese Medicine, Tianjin, China; 5 Tianjin University of Traditional Chinese Medicine, Tianjin, China

**Keywords:** acupuncture combined with antidepressants, mild-to-moderate depressive disorder, systematic review, meta-analysis, trial sequential analysis

## Abstract

**Objective:**

To assess the synergistic effect of acupuncture combined with antidepressants in the treatment of mild-to-moderate depressive disorders.

**Methods:**

Our systematic search identified randomized controlled trials evaluating acupuncture combined with antidepressants for mild-to-moderate depression across eight databases, with records retrieved from each database’s establishment until October 29, 2025. Independent researchers critically reviewed the literature, recorded relevant data, and assessed the quality of research. Data were analyzed using RevMan 5.4, Stata 17.0, and TSA 0.9.5.10.

**Results:**

The study included a total of 975 patients across 15 trials. Meta-analysis revealed that compared with antidepressants alone, acupuncture combined with antidepressants could significantly improve patients’ HAMD-24 scores (*MD* = −1.43, 95% CI [−1.88, −0.98], *p* < 0.00001), HAMD-17 scores (*MD* = −2.80, 95% CI [−3.97, −1.62], *p* < 0.00001), early efficacy (*MD* = −2.00, 95% CI [−2.62, −1.38], *p* < 0.00001), total effective rate (*MD* = 2.44, 95% CI [1.65, 3.63], *p* < 0.00001), SDS scores (*MD* = −4.16, 95% CI [−5.70, −2.62], *p* < 0.00001), TESS scores (*MD* = −3.63, 95% CI [−5.50, −1.76], *p* = 0.0001) as well as the SERS scores (*MD* = −3.01, 95% CI [−3.79, −2.23], *P*<0.00001). Although there is publication bias in HAMD-24 and total effective rate, the trim-and-fill test has confirmed the robustness of the results. Trial sequential analysis (TSA) results demonstrated that acupuncture combined with antidepressants was significantly superior to antidepressants alone in improving HAMD-24 scores, HAMD-17 scores, early efficacy, total effective rate, SDS scores, TESS scores as well as the SERS scores. Moreover, TSA confirmed that the sample sizes for all outcomes were sufficient to support the robustness of these conclusions.

**Conclusion:**

Acupuncture combined with antidepressants demonstrates a clear synergistic effect in treating mild to moderate depression. The combined therapy not only significantly outperformed antidepressants alone on primary efficacy endpoints but also demonstrated early therapeutic advantages as early as one week post-treatment, while markedly reducing medication-related side effects.

**Systematic review registration:**

https://www.crd.york.ac.uk/PROSPERO/view/CRD42025641858, Identifier CRD42025641858.

## 1 Introduction

Depression, characterized by depressed mood, loss of interest, and lack of energy, is one of the most common mental disorders. Studies indicate that the global prevalence of depression is 28.4% in the elderly (1), and as high as 33.6% among college students (2), which seriously affects the quality of personal survival, and behaviors such as self-injury and suicide can also result in a shortened life expectancy (3). The use of antidepressants is currently the main treatment method to improve the symptoms of depression, which is often combined with the use of sedative-hypnotic drugs, antipsychotics (4); however, it commonly leads to adverse effects such as nausea, fatigue, and gastrointestinal and cardiovascular reactions (5). A cohort study based on the Q Research database found that antidepressant safety was 54.5% when antidepressants were used by depressed patients for 2 months, and by 12 months, safety was only 28.8% (6). Additionally, discontinuation symptoms such as nausea, dizziness, and mood swings occurred in approximately 31% of patients after discontinuing antidepressant medications (7).

Xu et al. (8) conducted a systematic evaluation of 16 studies were systematically evaluated and meta-analyzed, and the results indicated that acupuncture as an adjunctive therapy to antidepressants may improve treatment outcomes and reduce adverse drug reactions in patients receiving antidepressants, but it neither assessed whether the included studies had sufficient sample sizes to support the conclusions, nor did it qualify depression severity. Whereas mild-to-moderate depression accounts for a high proportion of depressed patients, reaching 80% (9), timely and effective interventions in the early stages of the disease may prevent further exacerbation or relapse. Therefore, this study integrates and summarizes relevant literature on acupuncture combined with antidepressants for treating mild to moderate depression, systematically evaluating the synergistic effects of their combined use against conventional antidepressant therapy as a control. To further reduce the risk of random error arising from limited sample size and multiple significance tests, we introduced sequential analysis of trials (TSA), thereby providing more reliable evidence-based support for high-quality clinical practice and decision-making. It is worth emphasizing that this study deliberately restricted the participant population to patients with mild to moderate depression. This design ensures that the findings provide more precise guidance for clinical early intervention. Additionally, we conducted a systematic assessment of treatment efficacy during the early therapeutic phase, introducing the “HAMD score at 1 week post-treatment” as an independent outcome measure for meta-analysis to quantify the initial therapeutic effects of combination therapy.

## 2 Methods

### 2.1 Search strategy

The search was conducted by two evaluators using the following databases: (1) PubMed, Embase, Cochrane Central Register of Controlled Trials database (CENTRAL) and Web of Science (WOS); and (2) China National Knowledge Infrastructure (CNKI), VIP Database, WF Database, and SinoMed Database (CBM). We searched for studies published from the inception of the databases to October 29, 2025. A three-part search strategy was employed based on disease identification (mild-to-moderate depressive disorder), intervention analysis (acupuncture), and study design classification (RCT). Detailed search strategy is presented in Supplementary file 1. The program has been registered with PROSPERO. The registration number was CRD42025641858.

### 2.2 Inclusion criteria

#### 2.2.1 Participants

1. The age of patients is not required, but excludes studies on special groups (e.g., menopause, postpartum, the elderly, etc.).
2. Met at least one of the following diagnostic criteria: (i) World Health Organization Catalog of Mental and Behavioral Disorders (ICD-10); (ii) Chinese Classification and Diagnostic Criteria for Mental Disorders, 3rd edition (CCMD-3); (iii) United States Diagnostic Statistical Manual of Mental Disorders, Fourth Edition (DSM-IV); (iv) United States Diagnostic Statistical Manual of Mental Disorders, Fifth Edition (DSM-V).
3. The basis for judging the degree of mild-to-moderate depression was 20–35 points on the HAMD-24 or 17–24 points on the HAMD-17.
4. There was no explicit requirement for the sample size.

#### 2.2.2 Interventions

The control group was treated with pure antidepressants; the experimental group was treated with antidepressants combined with acupuncture or electroacupuncture or transcutaneous acupoint electrical stimulation (excluding ear acupuncture, abdominal acupuncture, acupoint injection and other special needling methods), and the antidepressants should be consistent with the control group. The acupoints and needle manipulation methods were not required.

#### 2.2.3 Outcomes

Outcome indicators included Hamilton Depression Scale (HAMD-24 or HAMD-17) and any of the following:

1. Total effective rate;
2. Self-rating depression scale (SDS);
3. The Treatment Emergent Symptom Scale (TESS);
4. Side Effects Rating Scale for Antidepressants (SERS).

#### 2.2.4 Research type

Randomized controlled trials (RCTs) were included. Studies with complete reporting of group sample sizes and outcome measures.

#### 2.2.5 Exclusion criteria

1. Participants suffered from other serious illnesses, such as suffering from severe aphasia, combined with severe organ damage, or with vascular dementia, or patients with bipolar disorder depressive episodes.
2. Duplicated publication.
3. Studies that combined several therapies were superior to acupuncture therapy alone.
4. Acupuncture therapy for special populations, such as the elderly, menopause, pregnant women, and children.
5. Studies lacking critical data that could not be extracted or accessed.

### 2.3 Literature screening and data extraction

Literature screening was conducted independently by two researchers. The core criterion for inclusion decisions was the severity of depression during the baseline period, specifically defined as: HAMD-24 scores ranging from 20 to 35, or HAMD-17 scores ranging from 17 to 24. Studies that specified only a lower score threshold but explicitly excluded severe depression in their text were also included, given that their patient population closely aligned with this study’s “mild to moderate” objective. The first author, year of publication, country of origin, participants’ characteristics (age and sex), intervention details, control group information, duration of the intervention, and outcome indicators were systematically tabulated. The selection and extraction of data were conducted independently by two researchers in accordance with the PRISMA guidelines. In case of any disagreement regarding data statistics, a third investigator was involved for assessment.

### 2.4 Study risk of bias assessment

The risk of bias in the studies was independently assessed by two investigators using the Cochrane Collaboration’s risk of bias tool (ROB) in RevMan 5.4 software. If there was disagreement between two investigators’ assessments, a third investigator performed the assessment. Each item was categorized as having low, high, or unclear risk of bias.

### 2.5 Data analysis

The meta-analysis was performed using RevMan 5.4 software. Continuous variables were expressed as mean difference (MD), and effect sizes along with their corresponding 95% confidence intervals were based on post-treatment means. Heterogeneity of effect sizes was assessed using the *I*^2^ index. A fixed-effect model was employed when heterogeneity was low (*I*^2^ < 50%, *p* ≥ 0.1). In cases of high heterogeneity (*I*^2^ ≥ 50%, *p* < 0.1), a random-effects model was used, and sensitivity analyses meta regression were performed with Stata 17.0 software for further investigation.

Additionally, funnel plots were generated once the number of included RCTs reached 10. Egger’s test was used to examine potential reporting bias in Stata 17.0.

Trial sequential analysis (TSA) was conducted using TSA 0.9.5.10 (Copenhagen Trial Unit, Denmark).^1^ The sample size was used as the expected information value, and the analysis was performed with a significance level of *α* = 0.05 for type I error, power of *β* = 0.2 for type II error, and a statistical efficacy of 80% to minimize random errors and ascertain the reliability of the results. Additionally, an estimation of the required sample size for meta-analysis was also carried out.

## 3 Results

### 3.1 Study selection

**Figure 1:**
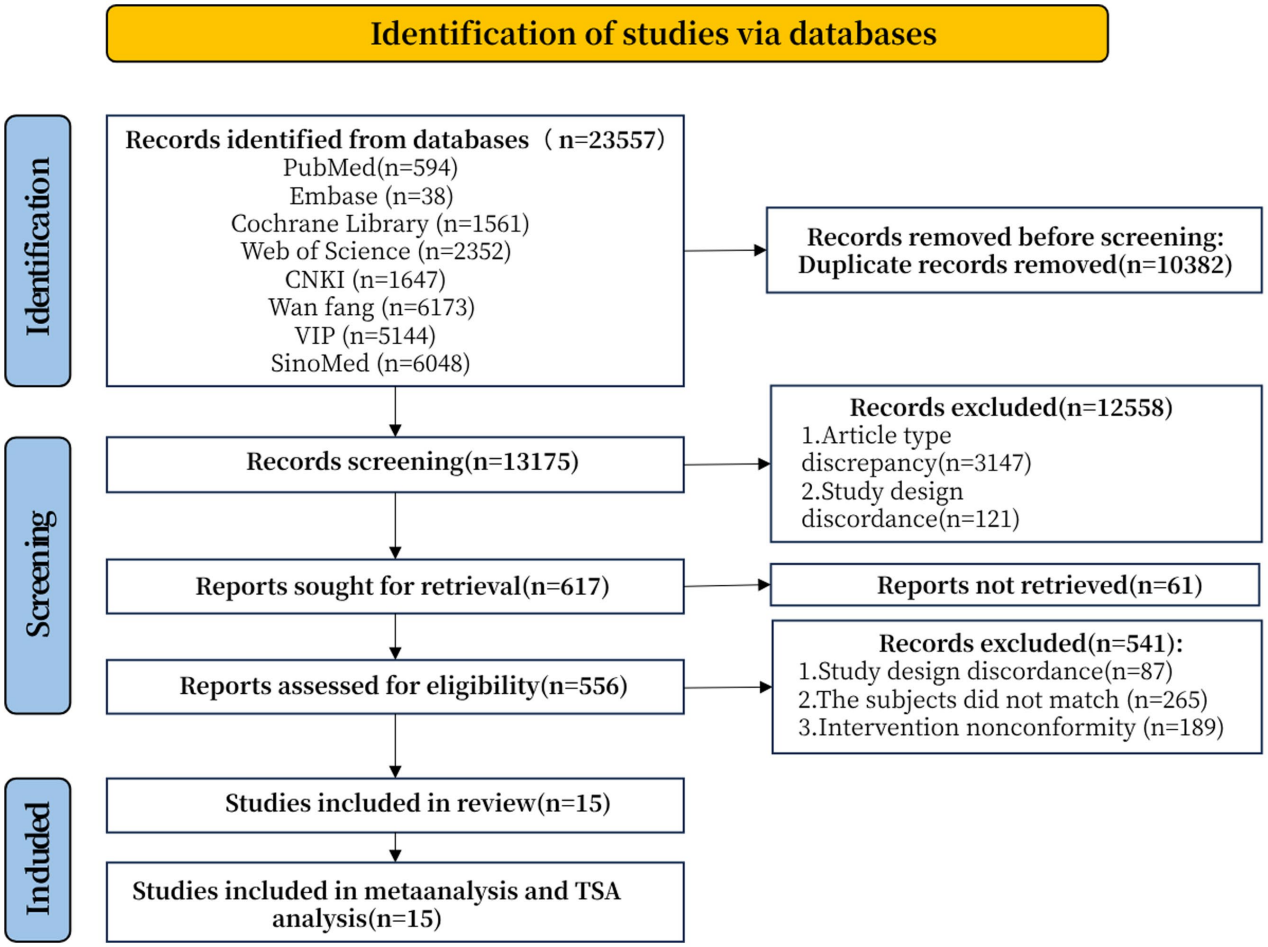
PRISMA flow diagram of included articles.

A total of 23,557 potential articles were initially identified from database searches and manual retrieval. After duplicates were removed, 13,175 articles were screened by examining their titles, abstracts, and keywords. The full text of 617 studies was further evaluated, and 602 articles were excluded for failing to meet the inclusion criteria. Finally, 15 studies were included in our systematic review and meta-analysis. Details of the study selection process are summarized in Figure 1.

### 3.2 Characteristics of included studies

This study encompassed 15 papers [3 studies (10–12) published in English and 12 studies (13–24) published in Chinese]. The collective sample size comprised 975 participants, with 504 assigned to the experimental group and 471 to the control group.

Manual acupuncture was used in nine studies (12, 13, 17–23), while electroacupuncture was used in six studies (10, 11, 14–16, 22). Ten studies (10, 11, 13–15, 17–20, 23) used HAMD-24, five studies (12, 16, 21, 22) used HAMD-17, and three studies (12, 14, 18) used SDS to assess patients’ depressive symptoms. Seven studies (13, 16, 18–20, 22) reported the severity of depression after 1 week of treatment. 12 studies (10, 12–16, 18–20, 22, 23) used total effective rate to assess patients’ clinical effectiveness. Four studies (10, 15, 21, 23) evaluated patients’ drug side effects using TESS, while six studies (13, 16, 19, 20, 22) using SERS. A comprehensive list of the included studies is provided in Table 1.

**Table 1:** Basic information of the included literature.

Studies	Sample size (T/C)	Randomization	Gender (M/F)	Average age (years)	Average duration of illness (months)	Intervention	Treatment duration (weeks)	Western diagnostic criteria	Outcomes
Cai et al. (13)	41/41	Random number table	T: 13/28	T: 45.27 ± 13.3	T: 23.51 ± 12.19	T: MA + SSRIs	6	③	①④⑥
			C: 14/27	C: 46.23 ± 14.5	C: 23.92 ± 14.52	C: SSRIs			
Duan and Tu (14)	20/20	NR	NR	NR	NR	T: EA + fluoxetine hydrochloride	6	①	①③④
						C: fluoxetine hydrochloride			
Duan et al. (10)	48/47	Random number table	T: 17/31	T: 38 ± 10.17	T: 16.2 ± 8.4	T: EA + fluoxetine hydrochloride	6	②	①④⑤
			C: 19/28	C: 37 ± 11.22	C: 17.9 ± 4.8	C: fluoxetine hydrochloride			
Duan et al. (15)	25/25	Random number table	T: 5/20	T: 48.93 ± 7.60	T: 18.33 ± 4.34	T: EA + fluoxetine hydrochloride	6	①	①④⑤
			C: 4/21	C: 49.72 ± 5.47	C: 17.92 ± 4.78	C: fluoxetine hydrochloride			
Li et al. (11)	30/30	Random number table	T: 11/19	T: 40.3 ± 10.99	NR	T: EA + antidepressants	8	③	①
			C: 11/19	C: 38.75 ± 11.45		C: antidepressants			
Ma et al. (16)	25/28	Centre randomized	NR	T: 46.27 ± 13.13	NR	T: EA + paroxetine hydrochloride	6	②	②④⑥
				C: 40.52 ± 14.21		C: paroxetine hydrochloride			
Shi et al. (17)	30/30	NR	T: 7/23	T: 43.65 ± 10.59	T: 17.45 ± 10.38	T: MA + escitalopram	4	②	①
			C: 8/22	C: 42.90 ± 14.32	C: 17.50 ± 11.68	C: escitalopram			
Sun et al. (18)	41/41	Random number table	T: 16/25	T: 49 ± 11	T: 4.7 ± 2.0	T: MA + sertraline hydrochloride	6	②	①③④
			C: 14/27	C: 46 ± 12	C: 4.9 ± 1.9	C: sertraline hydrochloride			
Tao (19)	35/35	Random number table	T: 14/21	T: 42.67 ± 11.17	T: 12.19 ± 4.33	T: MA + paroxetine hydrochloride	6	②	①④⑥
			C: 11/24	C: 40.54 ± 11.63	C: 12.12 ± 4.29	C: paroxetine hydrochloride			
Tao (20)	32/32	Random number table	T: 14/18	T: 43.67 ± 11.06	T: 14.13 ± 8.43	T: MA + paroxetine hydrochloride	6	②	①④⑥
			C: 11/21	C: 41.54 ± 10.62	C: 14.16 ± 8.59	C: paroxetine hydrochloride			
Tian et al. (21)	25/25	Random number table	T: 14/11	T: 36.2 ± 4.5	T: 21.4 ± 5.5	T: MA + sertraline hydrochloride	14	④	②⑤
			C: 12/13	C: 35.4 ± 5.6	C: 20.8 ± 6.7	C: sertraline hydrochloride			
Wang et al. (22)	20/8	Centre randomized	NR	T: 47 ± 11	NR	T: EA + paroxetine hydrochloride	6	②	②④⑥
				C: 48 ± 9		C: paroxetine hydrochloride			
Wang et al. (12)	80/80	NR	T: 22/58	T: 47.9 ± 6.5	NR	T: MA + fluoxetine hydrochloride	8	④	②③④
			C: 58/52	C: 46.3 ± 7.2		C: fluoxetine hydrochloride			
Wang et al. (22)	27/8	Centre randomized	NR	T: 45 ± 12	NR	T: MA + paroxetine hydrochloride	6	②	②④⑥
				C: 48 ± 9		C: paroxetine hydrochloride			
Xi et al. (23)	34/34	Random number table	T: 12/22	T: 37.5 ± 4.15	T: 8.65 ± 1.28	T: MA + duloxetine hydrochloride	12	①	①④⑤
			C: 20/14	C: 37.42 ± 4.11	C: 8.50 ± 1.18	C: duloxetine hydrochloride			

T, treatment group; C, control group; NR, not report; Western diagnostic standards: ① CCMD-3, ② ICD-10, ③ DSM-IV, ④ DSM-V. Outcome indicators: ① HAMD-24, ② HAMD-17, ③ SDS, ④ total effective rate, ⑤ TESS, ⑥ SERS.

### 3.3 Risk of bias assessment

**Figure 2:**
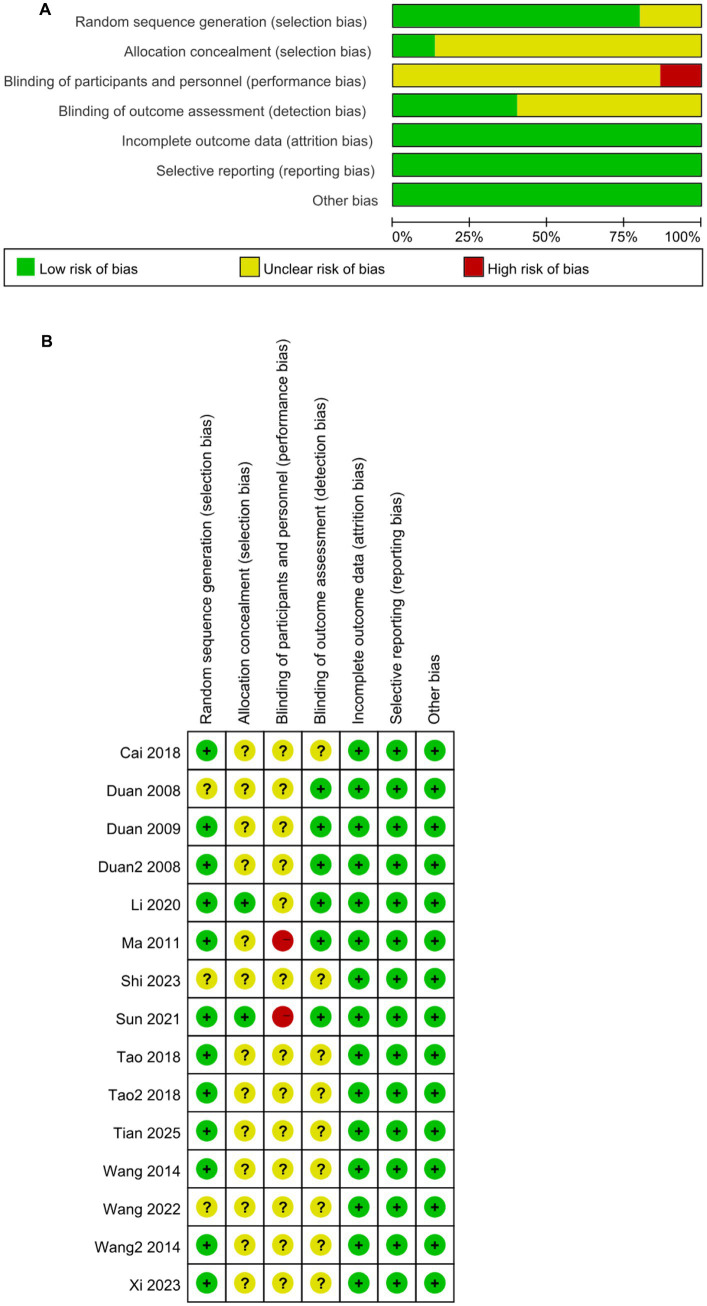
**(A)** Assessment of risk of bias presented as percentages across all included studies. **(B)** Risk of bias summary for each included study.

In terms of sequence generation, three studies (12, 14, 17) were rated as ‘unclear risk’ due to insufficient description of the randomization method. Two studies (16, 22) employed a centralized randomization system (low risk), and the remaining nine studies used random number tables (low risk). For allocation concealment, two studies (11, 18) implemented opaque envelopes (low risk), while the remaining studies did not provide sufficient information to assess this criterion (unclear). Regarding blinding of participants and personnel, two studies (16, 18) did not blind therapists (high risk), while the rest did not clearly state whether blinding was performed or not. In terms of blinding of outcome assessment, six studies (10, 11, 14–16, 18) blinded outcome evaluators (low risk), but for the rest of the studies it was unclear if blinding was conducted. Regarding the completeness of the outcome data, seven studies (10, 13–16, 18, 22) provided reasons for participant dropouts or missing data (low risk), whereas the remaining seven studies had no participant detachment mentioned (low risk). For selective reporting, all studies reported the prespecified outcome indicators (low risk). Regarding other biases, one study (14) reported balanced baseline information for both groups although baseline information was not specifically described; therefore, all studies clearly described the intervention protocol, data analysis methods, and balanced baseline information for both groups (low risk). Risk assessment results are presented in Figure 2.

## 4 Outcomes

### 4.1 HAMD-24

**Figure 3:**
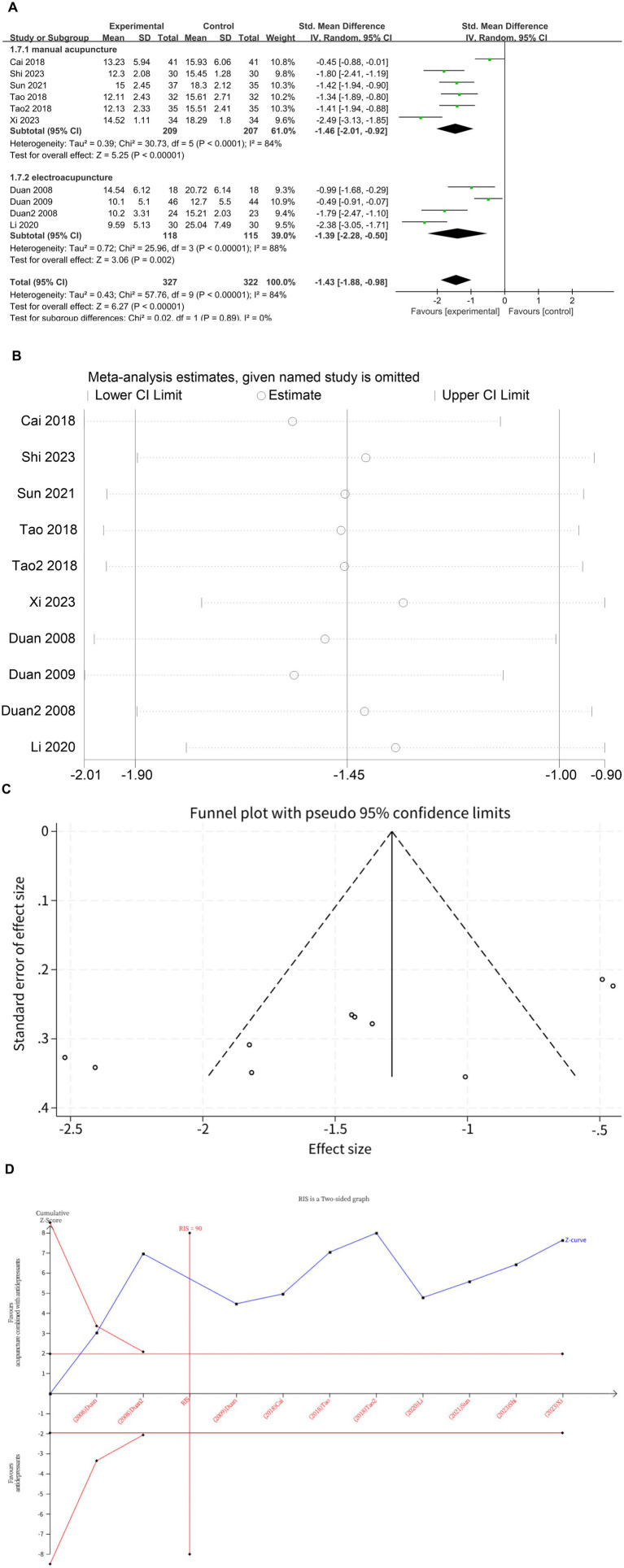
**(A)** Forest plot of HAMD-24 scale scores. **(B)** Sensitivity analysis of HAMD-24 scale scores. **(C)** A funnel plot of HAMD-24 scale scores. **(D)** Trial sequential analysis of HAMD-24 scale scores.

Ten studies (10, 11, 13–15, 17–20, 23) investigated changes in HAMD-24 before and after treatment, with a total of 649 subjects enrolled. The studies exhibited substantial heterogeneity (*I*^2^ = 84%, *p* < 0.00001), which was analyzed using random effects modeling. Acupuncture combined with antidepressants demonstrated statistically significant improvement in patients’ depressive symptoms compared to antidepressants alone (*MD* = −1.43, 95%CI [−1.88, −0.98], *p* < 0.00001; Figure 3A). Sensitivity analysis confirmed the stability of the results (Figure 3B). Subgroup analysis according to acupuncture modality showed that manual acupuncture combined with antidepressants (*MD* = −1.46, 95%CI [−2.01, −0.92], *p* < 0.00001) and electroacupuncture combined with antidepressants (*MD* = −1.39, 95%CI [−2.28, −0.50], *p* = 0.002) were superior to antidepressants alone in improving HAMD-24 scores (Figure 3A). The meta-regression analysis suggested that neither the type of acupuncture intervention (*p* = 0.703), treatment duration (*p* = 0.156), treatment frequency (*p* = 0.997) nor needle retention time (*p* = 0.758) appeared to be major contributors to the observed heterogeneity. Therefore, the heterogeneity may be attributable to difficult-to-quantify clinical factors within the included studies, such as acupuncturist technique, individual patient response, and acupoint selection. The funnel plot showed an asymmetrical distribution of combined effect sizes from the ten studies on HAMD-24 (Figure 3C), suggesting potential publication bias, which was subsequently confirmed by Egger’s test (*p* = 0.004 < 0.05). The trim-and-fill test filled in three studies and improved funnel plot symmetry, without altering the results, indicating that findings remain robust despite publication bias. The TSA demonstrated that the cumulative Z-curve had crossed the RIS boundary (RIS = 90) for the improvement in HAMD-24 scores, indicating that the required sample size had been reached and the evidence was sufficient. (Figure 3D).

### 4.2 HAMD-17

**Figure 4:**
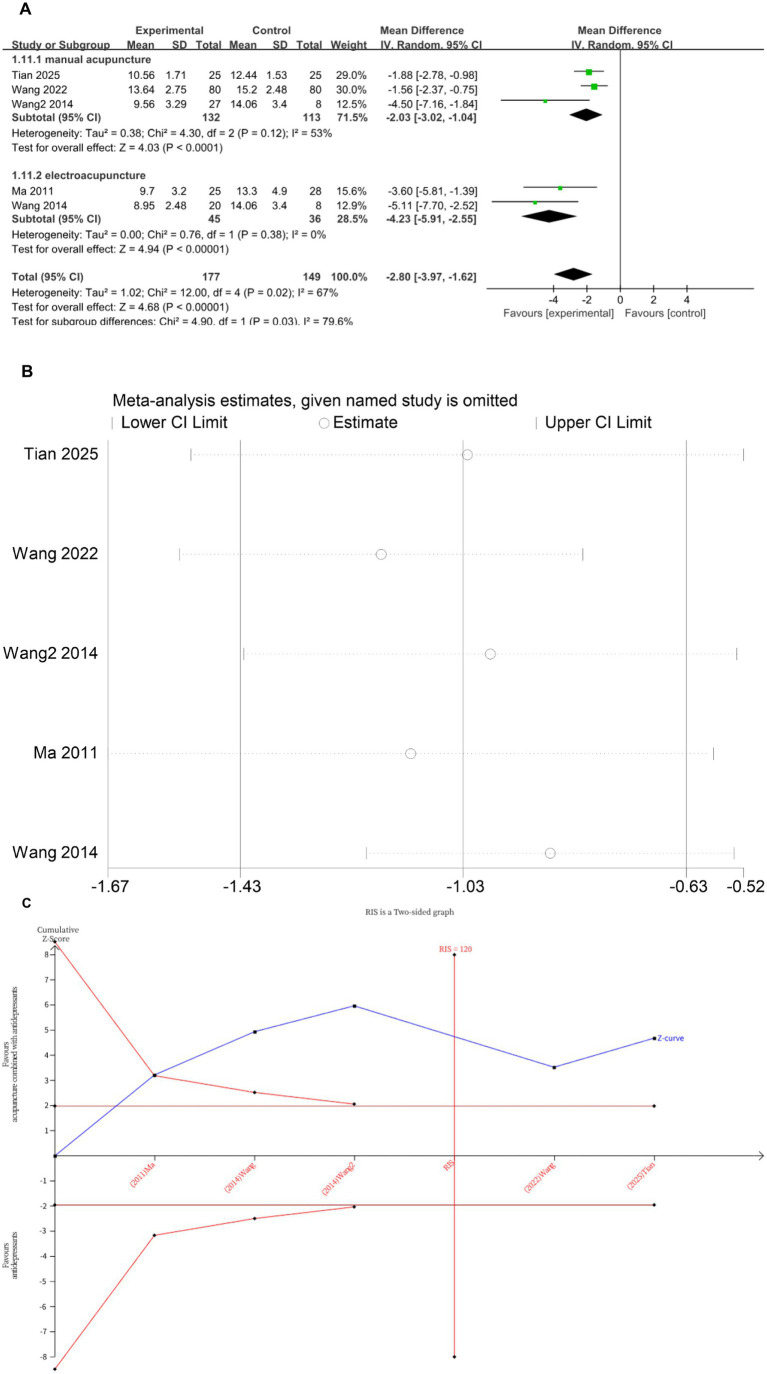
**(A)** Forest plot of HAMD-17 scale scores. **(B)** Sensitivity analysis of HAMD-17 scale scores. **(C)** Trial sequential analysis of HAMD-17 scale scores.

Five studies (12, 16, 21, 22) investigated changes in HAMD-17 before and after treatment, with a total of 326 subjects enrolled. The studies exhibited substantial heterogeneity (*I*^2^ = 67%, *p* = 0.02), which was analyzed using random effects modeling. Acupuncture combined with antidepressants demonstrated statistically significant improvement in patients’ depressive symptoms compared to antidepressants alone (*MD* = −2.80, 95%CI [−3.97, −1.62], *p* < 0.00001; Figure 4A). Sensitivity analysis confirmed the stability of the results (Figure 4B). Subgroup analysis according to acupuncture modality showed that manual acupuncture combined with antidepressants (*MD* = −2.03, 95%CI [−3.02, −1.04], *p* < 0.0001) and electroacupuncture combined with antidepressants (*MD* = −4.23, 95%CI [−5.91, −2.55], *p* < 0.00001) were superior to antidepressants alone in improving HAMD-17 scores (Figure 4A). The meta-regression analysis suggested that treatment frequency was a significant source of heterogeneity (*p* = 0.017). The TSA demonstrated that the cumulative Z-curve had crossed the RIS boundary (RIS = 120) for the improvement in HAMD-17 scores, indicating that the required sample size had been reached and the evidence was sufficient (Figure 4C).

### 4.3 Early efficacy (HAMD scores at 1 week of treatment)

**Figure 5:**
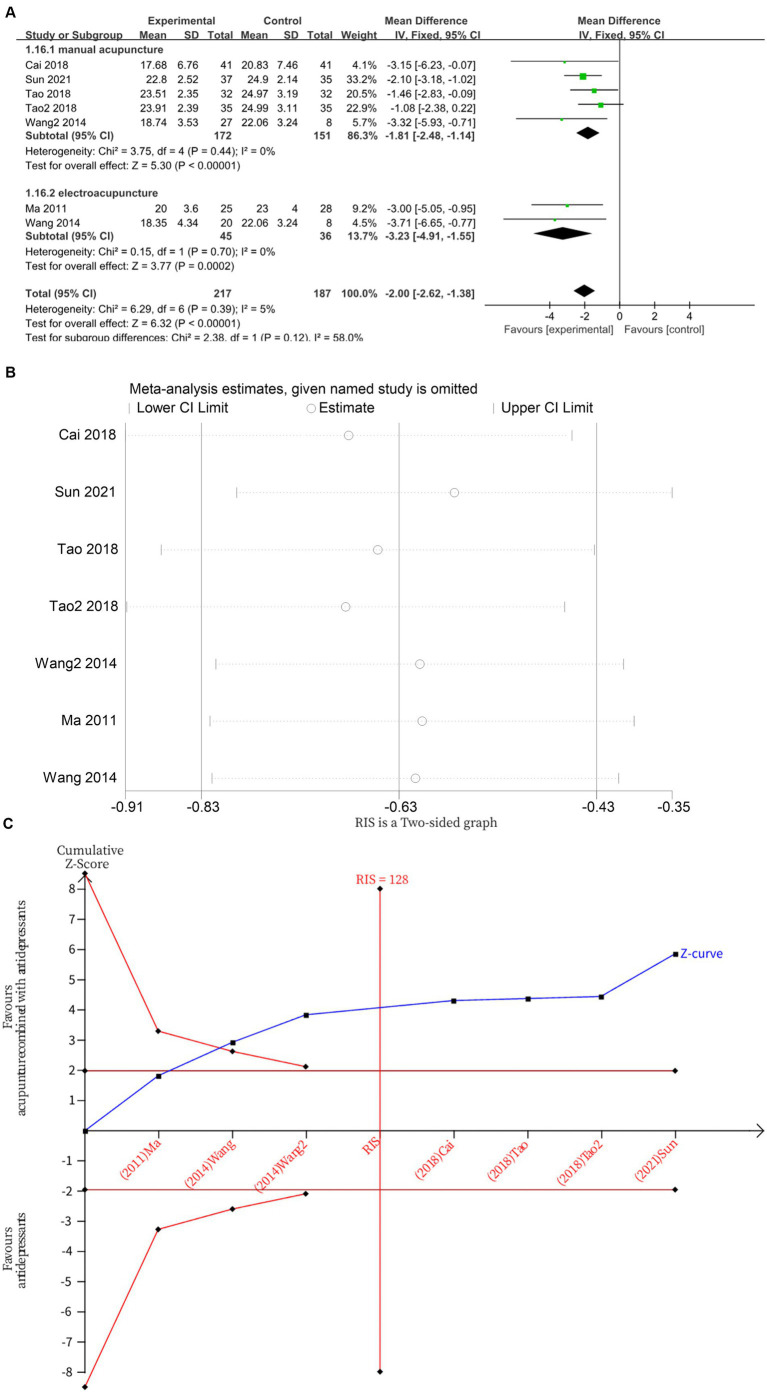
**(A)** Forest plot of HAMD scores at 1 week of treatment. **(B)** Sensitivity analysis of HAMD scores at 1 week of treatment. **(C)** Trial sequential analysis of HAMD scores at 1 week of treatment.

Seven studies (13, 16, 18–20, 22) investigated changes in HAMD before and one week after treatment, with a total of 404 subjects enrolled. The studies did not exhibited heterogeneity (*I*^2^ = 5%, *p* = 0.39), which was analyzed using fixed effects modeling. Acupuncture combined with antidepressants demonstrated statistically significant improvement in patients’ depressive symptoms after 1 week of treatment compared to antidepressants alone (*MD* = −2.00, 95%CI [−2.62, −1.38], *p* < 0.00001; Figure 5A). Sensitivity analysis confirmed the stability of the results (Figure 5B). Subgroup analysis according to acupuncture modality showed that, at 1 week, manual acupuncture combined with antidepressants (*MD* = −1.81, 95%CI [−2.48, −1.14], *p* < 0.00001) and electroacupuncture combined with antidepressants (*MD* = −3.23, 95%CI [−4.91, −1.55], *p* = 0.0002) were superior to antidepressants alone in improving HAMD scores (Figure 5A). The TSA demonstrated that the cumulative Z-curve had crossed the RIS boundary (RIS = 128) for the improvement in HAMD scores at 1 week, indicating that the required sample size had been reached and the evidence was sufficient (Figure 5C).

### 4.4 Total effective rate

**Figure 6:**
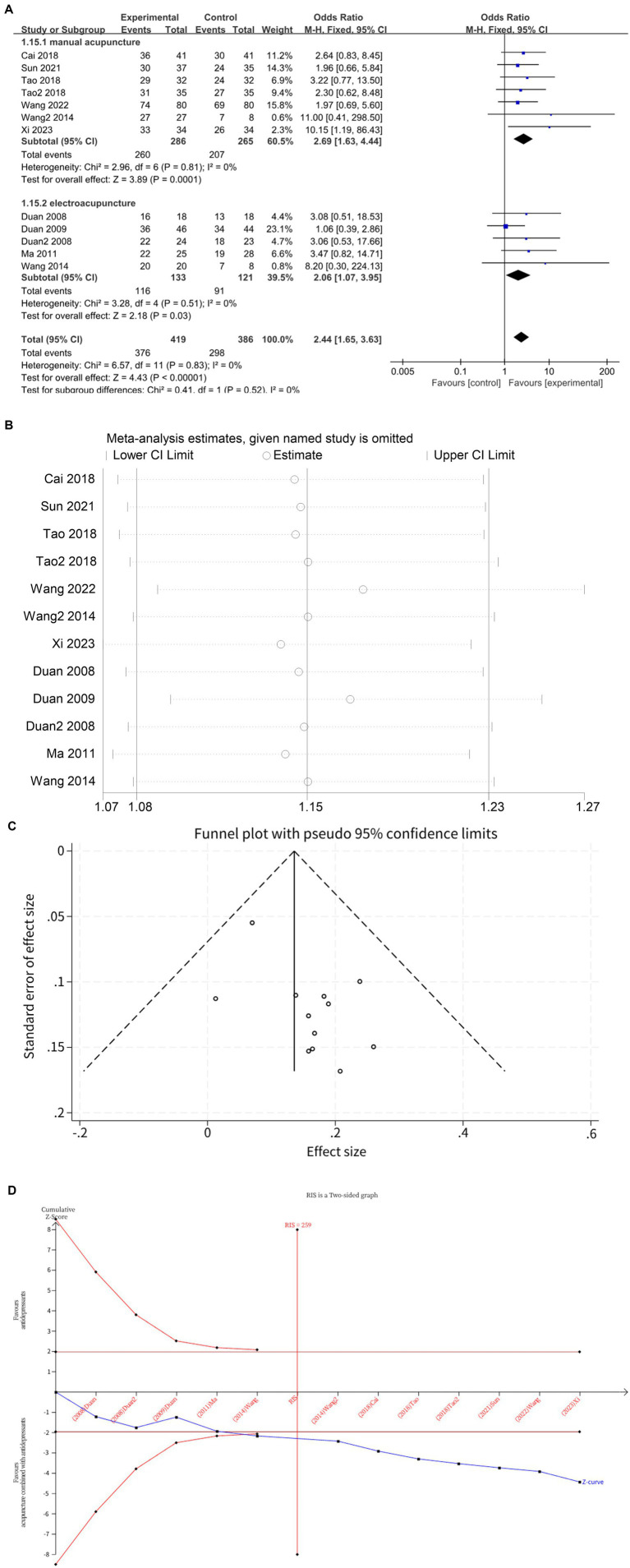
**(A)** Forest plot of total effective rate. **(B)** Sensitivity analysis of total effective rate. **(C)** A funnel plot of total effective rate. **(D)** Trial sequential analysis of total effective rate.

Twelve studies (10, 12–16, 18–20, 22, 23) investigated changes in total effective rate after treatment, with a total of 805 subjects enrolled. The studies did not exhibited heterogeneity (*I*^2^ = 0%, *p* = 0.83), which was analyzed using fixed effects modeling. Acupuncture combined with antidepressants demonstrated statistically significant improvement in patients’ total effective rate compared to antidepressants alone (*MD* = 2.44, 95%CI [1.65, 3.63], *p* < 0.00001) (Figure 6A). Sensitivity analysis confirmed the stability of the results (Figure 6B). Subgroup analysis according to acupuncture modality showed that manual acupuncture combined with antidepressants (*MD* = 2.69, 95%CI [1.63, 4.44], *p* = 0.0001) and electroacupuncture combined with antidepressants (*MD* = 2.06, 95%CI [1.07, 3.95], *p* = 0.03) were superior to antidepressants alone in improving total effective rate (Figure 6A). The funnel plot showed an asymmetrical distribution of combined effect sizes from the 12 studies on total effective rate, suggesting potential publication bias (Figure 6C), which was subsequently confirmed by Egger’s test (*p* = 0.031 < 0.05). The trim-and-fill test filled in five studies and improved funnel plot symmetry, without altering the results, indicating that findings remain robust despite publication bias. The TSA demonstrated that the cumulative Z-curve had crossed the RIS boundary (RIS = 259) for the improvement in total effective rate, indicating that the required sample size had been reached and the evidence was sufficient (Figure 6D).

### 4.5 SDS

**Figure 7:**
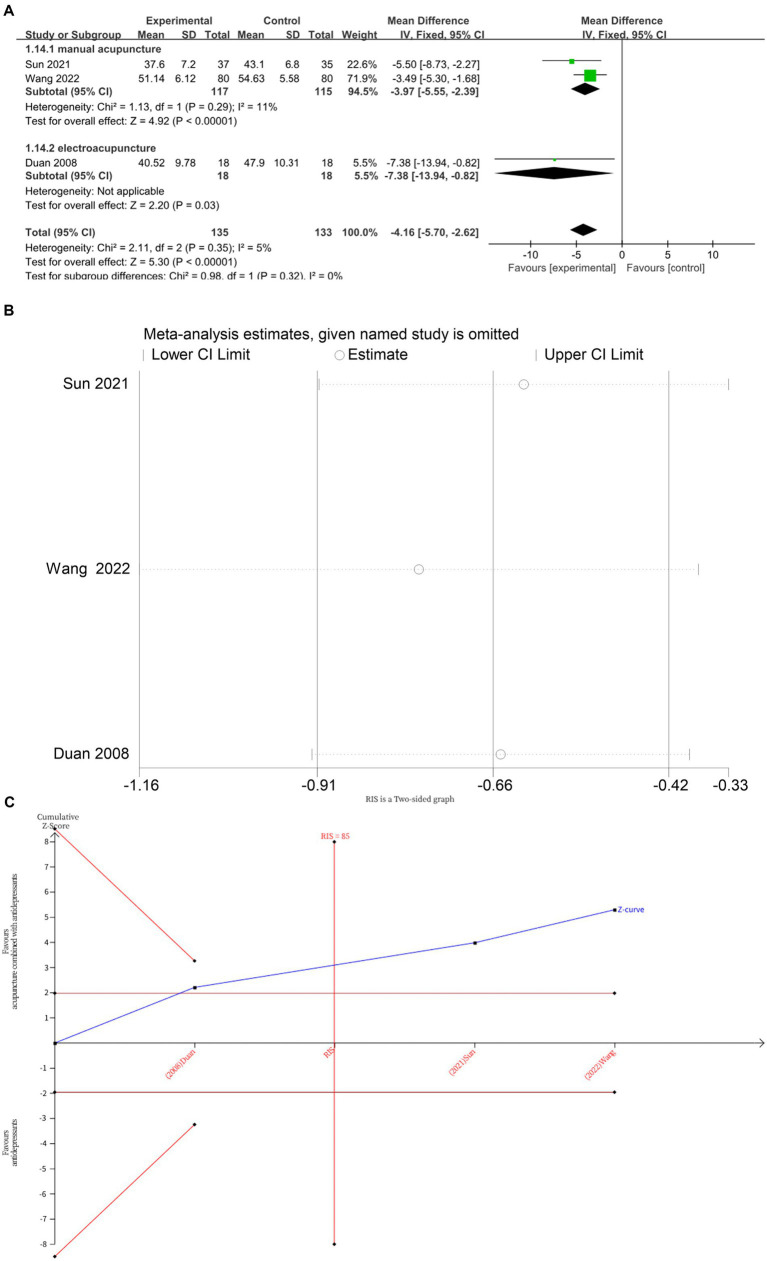
**(A)** Forest plot of SDS scale scores. **(B)** Sensitivity analysis of SDS scale scores. **(C)** Trial sequential analysis of SDS scale scores.

Three studies (12, 14, 18) investigated changes in SDS before and after treatment, with a total of 268 subjects enrolled. The studies did not exhibited heterogeneity (*I*^2^ = 5%, *p* = 0.35), which was analyzed using fixed effects modeling. Acupuncture combined with antidepressants demonstrated statistically significant improvement in patients’ depressive symptoms compared to antidepressants alone (*MD* = −4.16, 95%CI [−5.70, −2.62], *p* < 0.00001; Figure 7A). Sensitivity analysis confirmed the stability of the results (Figure 7B). Subgroup analysis according to acupuncture modality showed that manual acupuncture combined with antidepressants (*MD* = −3.97, 95%CI [−5.55, −2.39], *p* < 0.00001) and electroacupuncture combined with antidepressants (*MD* = −7.38, 95%CI [−13.94, −0.82], *p* = 0.03) were superior to antidepressants alone in improving SDS scores (Figure 7A). The TSA demonstrated that the cumulative Z-curve had crossed the RIS boundary (RIS = 85) for the improvement in SDS scores, indicating that the required sample size had been reached and the evidence was sufficient (Figure 7C).

### 4.6 Tess

**Figure 8:**
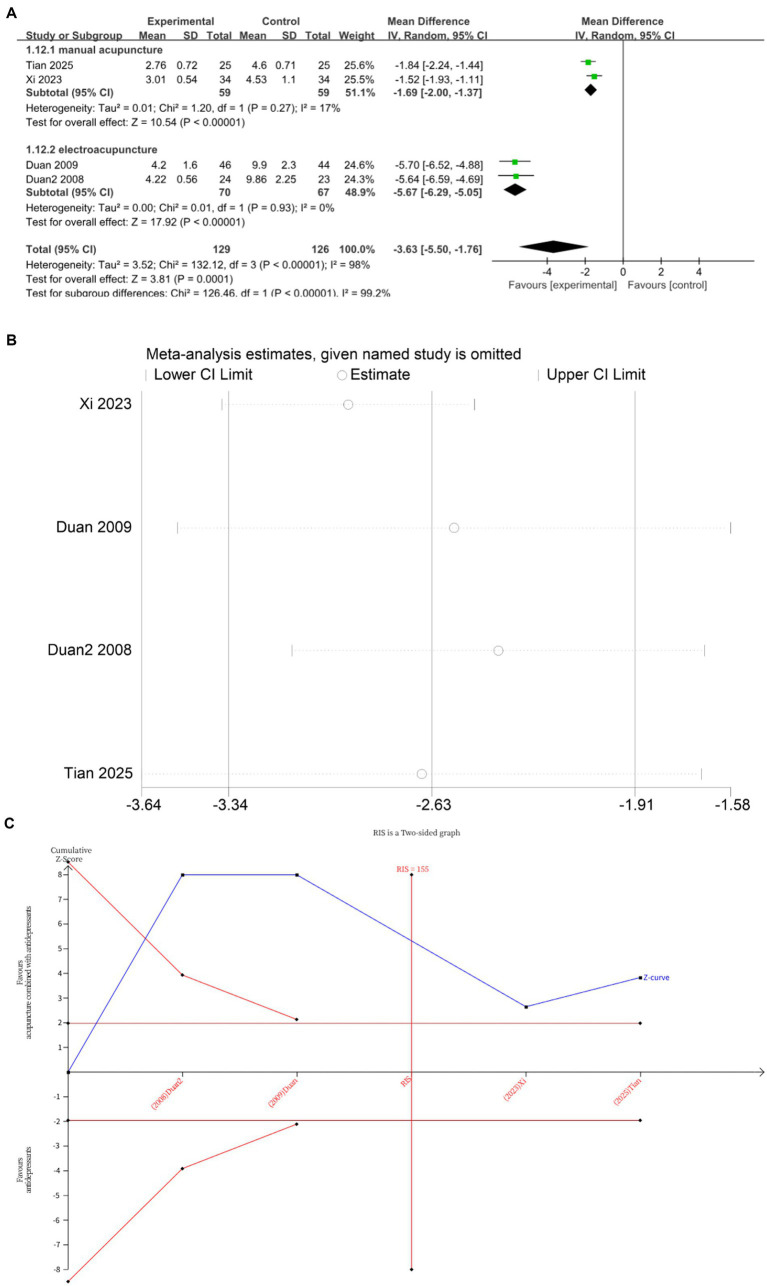
**(A)** Forest plot of TESS scale scores. **(B)** Sensitivity analysis of TESS scale scores. **(C)** Trial sequential analysis of TESS scale scores.

Four studies (10, 15, 21, 23) investigated changes in TESS before and after treatment, with a total of 255 subjects enrolled. The studies exhibited substantial heterogeneity (*I*^2^ = 98%, *p* < 0.00001), which was analyzed using random effects modeling. The addition of acupuncture significantly reduces the side effects of antidepressants, as evidenced by a decrease in TESS scores (MD = −3.63, 95%CI [−5.50, −1.76], *p* = 0.0001; Figure 8A). Sensitivity analysis confirmed the stability of the results (Figure 8B). Subgroup analysis according to acupuncture modality showed that both manual acupuncture (*MD* = −1.69, 95%CI [−2.00, −1.37], *p* < 0.00001) and electroacupuncture (*MD* = −5.67, 95%CI [−6.29, −5.05], *p* < 0.00001), when combined with antidepressants, significantly reduced the side effects of antidepressants, as evidenced by decreased TESS scores (Figure 8A). The meta-regression analysis suggested that the type of acupuncture intervention was a significant source of heterogeneity (*p* = 0.044 < 0.05). The TSA demonstrated that the cumulative Z-curve had crossed the RIS boundary (RIS = 155), indicating that the sample size was sufficient to conclude that acupuncture combined with antidepressants significantly reduced TESS scores (Figure 8C).

### 4.7 Sers

**Figure 9:**
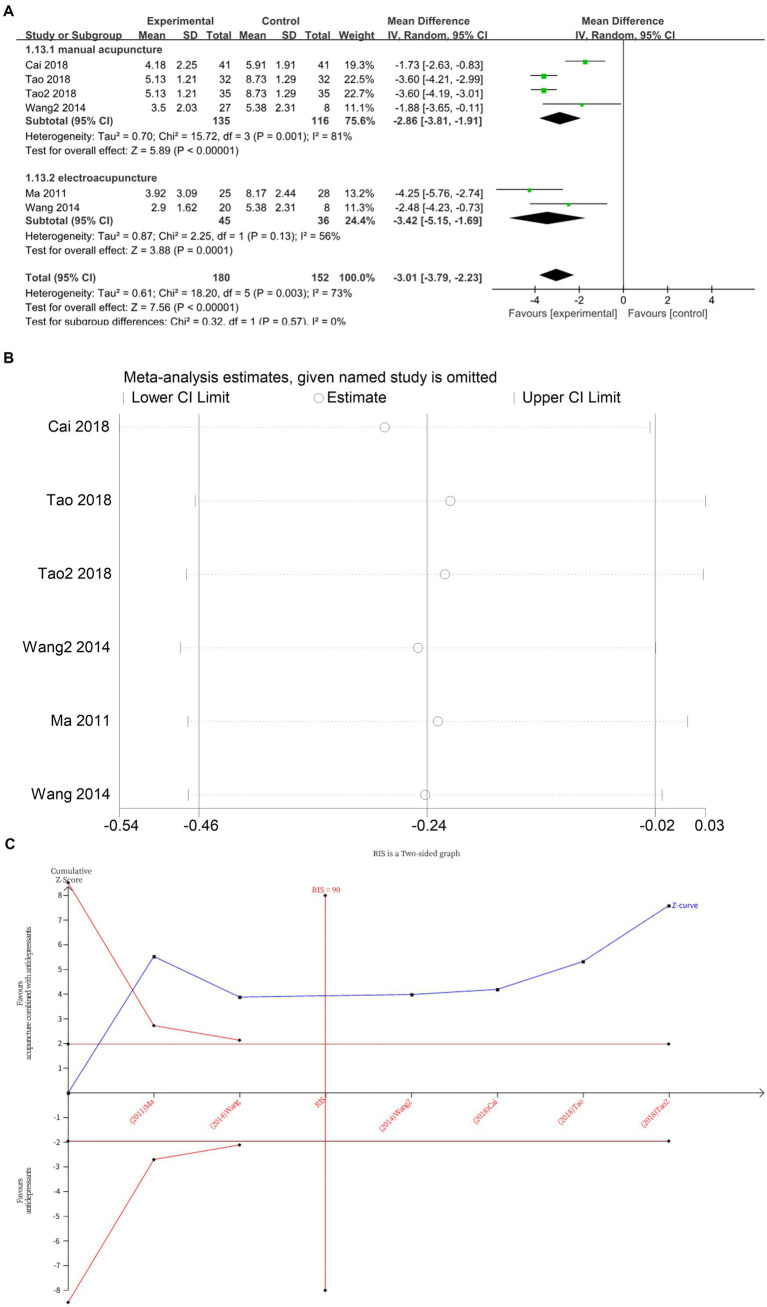
**(A)** Forest plot of SERS scale scores. **(B)** Sensitivity analysis of SERS scale scores. **(C)** Trial sequential analysis of SERS scale scores.

Six studies (13, 16, 19, 20, 22) investigated changes in SERS before and after treatment, with a total of 332 subjects enrolled. The studies exhibited substantial heterogeneity (*I*^2^ = 73%, *p* = 0.003), which was analyzed using random effects modeling. The addition of acupuncture significantly reduces the side effects of antidepressants, as evidenced by a decrease in SERS scores (*MD* = −3.01, 95%CI [−3.79, −2.23], *p* < 0.00001) (Figure 9A). Sensitivity analysis confirmed the stability of the results (Figure 9B). Subgroup analysis according to acupuncture modality showed that both manual acupuncture (*MD* = −2.86, 95%CI [−3.81, −1.91], *p* < 0.00001) and electroacupuncture (*MD* = −3.42, 95%CI [−5.15, −1.69], *p* = 0.0001), when combined with antidepressants, significantly reduced the side effects of antidepressants, as evidenced by decreased SERS scores (Figure 9A). The meta-regression analysis suggested that neither the type of acupuncture intervention (*p* = 0.776), treatment frequency (*p* = 0.497) nor the type of HAMD scale (*p* = 0.977) appeared to be major contributors to the observed heterogeneity. Therefore, the heterogeneity may be attributable to difficult-to-quantify clinical factors within the included studies, such as acupuncturist technique, individual patient response, and acupoint selection. The TSA demonstrated that the cumulative Z-curve had crossed the RIS boundary (RIS = 90), indicating that the sample size was sufficient to conclude that acupuncture combined with antidepressants significantly reduced SERS scores (Figure 9C).

## 5 Adverse event reporting

In seven studies (10, 13, 14, 16, 17, 22), adverse events have been reported, of which, four studies (13, 14, 16, 17) were related to acupuncture. The remaining nine studies did not report adverse events.

Cai et al. (13) and Ma et al. (16) each reported one case of termination of treatment due to inability to adhere to acupuncture.

Duan et al. (14) reported three cases of termination of treatment due to rash, postural hypotension, nervousness, insomnia, and headache, all of which were considered drug-induced side effects. In one case, treatment was terminated due to discomfort with the sensation of acupuncture.

Shi et al. (17) reported one case of a patient who experienced needle fainting during acupuncture, which improved with prompt symptomatic treatment and did not affect the overall treatment course.

Duan et al. (10) reported the termination of treatment in one patient due to dizziness and postural hypotension after 1 week of fluoxetine, in one patient due to panic and tachycardia on electrocardiogram after 4 weeks of fluoxetine, in one patient due to sexual dysfunction after taking the drug, and in one patient due to dysuria after taking the drug.

Wang et al. (22) reported one case of termination of treatment due to dizziness without specifying whether it was related to acupuncture or medication.

Overall, significantly more participants discontinued antidepressants than acupuncture during treatment, and acupuncture was associated with milder adverse events that did not affect subsequent treatment.

## 6 Discussion

Currently, clinical antidepressants mainly include: selective serotonin reuptake inhibitors (SSRIs), serotonin-norepinephrine reuptake inhibitors (SNRIs), norepinephrine reuptake inhibitors (NRIs), and other classes. These are often used in combination with sedative-hypnotics, antipsychotics, and other adjunctive medications. Statistically (24), the highest incidence of adverse reactions (45%) was observed with antidepressants combined with sedative-hypnotics, followed by combinations with antipsychotics (25%), combined sedative-hypnotics and antipsychotics (8%), and antidepressant monotherapy (8%). The therapeutic effects of antidepressants are mediated mainly through modulation of serotonin (5-HT) and dopamine neurotransmission, which simultaneously accounts for their adverse effect profile. The adverse effects of SSRIs stem from excessive stimulation of 5-HT receptors: overactivation of 5-HT2A/2C receptors induces sexual dysfunction, with reported incidence rates of 24–73% in SSRI-treated patients (25, 26); agonism at 5-HT3 receptors mediates nausea and vomiting (27); activation of 5-HT1A receptors enhances alertness while reducing REM sleep, resulting in sleep disturbances (28). Adverse effects induced by NRIs may result from excessive stimulation of noradrenergic receptor subtypes. Specifically, overactivation of α1-adrenergic receptors can produce anticholinergic-like effects, including dry mouth and constipation (29). In addition, it can also cause central nervous system side effects such as anxiety, dizziness, and insomnia, as well as cardiovascular reactions like tachycardia and arrhythmia. When applying antidepressants clinically, we should not only pursue efficacy but also consider safety as a key factor.

The findings of this study reveal multiple advantages of combining acupuncture with antidepressant therapy in clinical practice, centered on achieving synergistic “synergistic enhancement” and “reduction of toxicity.” Regarding clinical efficacy, all studies included in this meta-analysis reached a consistent conclusion: acupuncture combined with antidepressants significantly outperforms antidepressants alone in treating mild to moderate depression, as demonstrated by both patient self-report (SDS scores) and clinical observer-rated scales (HAMD scores). This indicates that the benefits of combined therapy are comprehensive and tangibly perceived by patients. The observed synergy may be explained by the multi-target mechanisms of acupuncture, which include: regulating monoamine and amino acid neurotransmitter levels (30, 31); promoting neurotrophic factor expression to alleviate symptoms (32); facilitating neuronal repair and regeneration while suppressing neuroinflammation (33); and correcting dysfunction of the hypothalamic–pituitary–adrenal axis (34). Furthermore, given the reciprocal exacerbation between sleep disorders and depression, acupuncture combined with medication not only directly alleviates core symptoms but may also indirectly enhance overall antidepressant efficacy by significantly improving patients’ sleep quality (35, 36). In summary, acupuncture does not merely add to the effects of medication; rather, it constitutes a multi-targeted synergistic and complementary therapeutic model. It exerts synergistic effects with antidepressants through multi-level regulation involving neurobiochemistry, neuroendocrinology, and neuroplasticity.

Regarding safety, 10 studies (10, 13, 15, 16, 18, 19, 21–23) consistently demonstrated that combination therapy significantly reduced scores on the Side Effects Rating Scale (SERS/TESS). This outcome strongly demonstrates that incorporating acupuncture not only fails to increase treatment risks but effectively alleviates common adverse reactions to antidepressants. This holds promise for enhancing long-term treatment adherence by improving patient tolerance. More clinically significant, the combined therapy exhibits potential for rapid onset of action. As demonstrated by studies from Cai et al. (13), Ma et al. (16), Sun et al. (18) and Tao (19), the combined therapy group exhibited significant therapeutic differences after just 1 week of treatment, whereas medication alone typically requires 2 weeks to take effect. This “rapid onset” effect means patients can find relief from distress sooner—crucial for preventing disease progression and theoretically enabling reduced cumulative drug exposure. Shorter onset times may optimize the duration and dosage required to achieve stable therapeutic effects, thereby indirectly lowering the risk of long-term medication-related side effects. In summary, acupuncture mitigates the adverse effects of antidepressants through two principal pathways: the reduction of cumulative drug exposure via early symptom control and the direct reduction of long-term medication side effects.

A previously published meta-analysis suggested that acupuncture as an adjunctive therapy to antidepressants may improve treatment outcomes and reduce adverse drug reactions (8). However, the study had two key limitations: it neither assessed whether the included studies had sufficient sample sizes to support the conclusions, nor did it qualify depression severity (most participants had mild-to-moderate depression, where early intervention could prevent disease progression or relapse). Therefore, our study specifically evaluates the synergistic effects of acupuncture combined with antidepressants for mild-to-moderate depression. We aim to determine whether the combined approach is superior to antidepressants alone in improving depressive symptoms, and reducing adverse drug reactions. Additionally, we employed trial sequential analysis to verify whether the current sample size supports robust conclusions. We deliberately restricted the study population to patients with mild to moderate disease. This design provides more precise guidance for clinical early intervention and the selection of stepwise treatment regimens. Furthermore, to mitigate the risk of false positives (Type I errors) that may arise from repeated data updates in traditional meta-analyses, we introduced sequential analysis of trials for this research topic. This confirmed the robustness and reliability of the current positive results, with sufficient sample size, thereby providing a higher level of evidence for this topic. Finally, we systematically evaluated treatment response in the early phase. We added a meta-analysis of “HAMD scores at 1 week post-treatment” as an independent outcome measure. Quantitative analysis confirmed that combination therapy yields significant advantages as early as 1 week into treatment. This discovery of “rapid onset of action” represents a key innovation of our study, providing new evidence for early intervention.

This study included 15 RCTs and systematically evaluated the clinical efficacy of acupuncture combined with antidepressants for treating mild to moderate depression. Results showed that compared with antidepressants alone, both manual acupuncture and electroacupuncture combined with antidepressants significantly improved depressive symptoms (as assessed by HAMD and SDS), reduced medication-related adverse reactions (as assessed by TESS and SERS), and enhanced clinical efficacy (early response rate and overall response rate). TSA demonstrated that the cumulative sample size had reached the required information volume, supporting the reliability of the findings. Significant heterogeneity was observed in some outcome measures (e.g., HAMD-24, HAMD-17, TESS, SERS) during analysis. Therefore, we systematically conducted subgroup analyses and meta-regression. We found that acupuncture frequency was a major source of heterogeneity in the HAMD-17 analysis, while acupuncture intervention type was a key factor in the TESS analysis. However, for HAMD-24 and SERS, despite examining multiple variables including publication year, intervention type, and frequency, no statistically significant source of heterogeneity was identified. We speculate that this residual heterogeneity may stem from difficult-to-quantify clinical factors within the included studies, such as acupuncturist technique, individual patient response, and acupoint selection. Notably, despite heterogeneity, all studies showed consistent effect directions. Using a random-effects model for meta-analysis yielded more conservative and reliable estimates, ensuring robust primary conclusions. Given that all studies were conducted in China, publication bias risk exists. Egger’s test for potential publication bias was performed on outcomes with ≥10 included studies, revealing significant effects for HAMD-24 (*p* = 0.004) and overall response rate (*p* = 0.031). Further sensitivity analysis using trim-and-fill methods was conducted. After filling three and five theoretical missing studies for the aforementioned indicators, respectively, funnel plot symmetry improved without altering the statistical conclusions. This further substantiates the robustness of the findings, which remain unaffected by potential publication bias.

This study has several limitations. First, all included trials were conducted in China, posing a risk of publication bias that may overestimate treatment effects and limiting the geographical generalizability and cross-cultural applicability of the findings. Future high-quality randomized controlled trials across diverse healthcare cultures are needed to validate the efficacy of this combination therapy. Second, due to the unique nature of acupuncture interventions, achieving perfect blinding for both practitioners and participants is inherently challenging, resulting in a high risk of bias across the included studies. Subsequent studies should adopt more rigorous control designs to enhance the verifiability of results. Finally, despite performing multivariate meta-regression analyses on measures like HAMD-24 and SERS (including publication year and acupuncture type), we could not fully explain the statistical sources of heterogeneity. Residual heterogeneity likely stems from difficult-to-quantify clinical factors such as acupuncturist experience, point selection, and manipulation techniques. Future efforts should promote standardization of clinical protocols and incorporate objective biological markers in high-quality RCTs to more accurately assess treatment efficacy.

In summary, this study confirms through meta-analysis and trial sequence analysis that acupuncture combined with antidepressants demonstrates a clear synergistic effect in treating mild to moderate depression. The combined therapy not only significantly outperformed antidepressants alone on primary efficacy endpoints but also demonstrated early therapeutic advantages as early as 1 week post-treatment, while markedly reducing medication-related side effects.

## Data Availability

The original contributions presented in the study are included in the article/Supplementary material, further inquiries can be directed to the corresponding author/s.

## Footnotes

1 http://www.ctu.dk.
