# Supplementary material for: Acupuncture combined with antidepressants for mild-to-moderate depressive disorders: a systematic review with meta-analysis and trial sequential analysis
Source: Front Neurol. 2025 Dec 12;16:1636589. doi: 10.3389/fneur.2025.1636589 (PMC12741110; doi:10.3389/fneur.2025.1636589)
Supplement: Supplementary file 1 [file Supplementary_file_1.pdf]

Supplementary Materials

supplementary file 1 Example search strategy— Pubmed

**PubMed**		
**Item**	Search strategies	n
**#1**	((((((((depression[MeSH Terms]) OR (depression[Title/Abstract])) OR (Depressive[Title/Abstract])) OR (Depressed[Title/Abstract])) OR (depression disorder[Title/Abstract])) OR (Depressive Symptom[Title/Abstract])) OR (Symptom, Depressive[Title/Abstract])) OR (Emotional Depression[Title/Abstract])) OR (Depression, Emotional[Title/Abstract])	668147
**#2**	((((((((((((((((((Acupuncture[MeSH Terms]) OR (Acupuncture[Title/Abstract])) OR (Pharmacopuncture[Title/Abstract])) OR (electroacupuncture[Title/Abstract])) OR (Acupuncture Treatment[Title/Abstract])) OR (Acupuncture Treatments[Title/Abstract])) OR (Treatment, Acupuncture[Title/Abstract])) OR (Therapy, Acupuncture[Title/Abstract])) OR (Pharmacoacupuncture Treatment[Title/Abstract])) OR (Treatment, Pharmacoacupuncture[Title/Abstract])) OR (Pharmacoacupuncture Therapy[Title/Abstract])) OR (Therapy, Pharmacoacupuncture[Title/Abstract])) OR (Acupotomy[Title/Abstract])) OR (transcutaneous electrical acupoint stimulation[Title/Abstract])) OR (electrical acupoint stimulation[Title/Abstract])) OR (transcutaneous electrical stimulation of acupoints[Title/Abstract])) OR (transcutaneous electrical acupuncture point stimulation[Title/Abstract])) OR (transcutaneous electric nerve stimulation[Title/Abstract])) OR (electroacupuncture[Title/Abstract])	44293
**#3**	(((Randomized Controlled Trial[MeSH Terms]) OR (Randomized Controlled Trial[Title/Abstract])) OR (Clinical research[Title/Abstract])) OR (RCT[Title/Abstract])	411553
**#4**	#1 AND #2 AND #3	594
